# Enhancing patient mobility following cesarean-delivery – the efficacy of an improved postpartum protocol assessed with pedometers

**DOI:** 10.1186/s12884-020-03046-z

**Published:** 2020-06-09

**Authors:** Hadas Ganer Herman, Masha Ben Zvi, Daniel Tairy, Ilia Kleiner, Noa Gonen, Limor Kuper Sason, Jacob Bar, Michal Kovo

**Affiliations:** Departments of Obstetrics and Gynecology, Edith Wolfson Medical Center, Holon, Israel, affiliated with the Sackler Faculty of Medicine, Tel Aviv University, Tel Aviv, Israel

**Keywords:** Cesarean-delivery, Pedometer, Analgesics, Steps

## Abstract

**Background:**

The incidence of thromboembolic complications is highest in the immediate postpartum period, especially following caesarean delivery (CD). Ambulation following CD is important in their prevention. We examined the effect of an educational protocol on patients’ mobility following CD, with the use of digital step counters (pedometers).

**Methods:**

Starting February 2018, we implemented an educational protocol at the maternity ward, which included nurses’ tutoring and subsequent patients’ education, regarding the importance of early ambulation. Following CD, ambulation was initiated 4 h following surgery (as compared to 6 h prior). Scheduled IV acetaminophen was administered at six-hour intervals for 48 h (as compared to only 24 h prior), while additional analgesics were given upon patient request. We compared maternal demographics, delivery and postpartum course between the pre-protocol group (*n* = 101) and the post-protocol group (*n* = 100). All patients were asked to wear pedometers for 48 h following the delivery to assess ambulation.

**Results:**

Patients’ demographics, surgical and post-partum course were non-significant between the groups, except for surgical length (48.5 ± 14.6 vs. 53.5 ± 15.3 min in the pre and post protocol groups, respectively, *p* = 0.02). The pre-protocol group was treated with more additional analgesics (p = 0.02). A higher number of steps was taken in the post-protocol group as compared to the pre-protocol group (4394 ± 2985 vs.3551 ± 2931, respectively *p* = 0.04). In a linear regression analysis in which the number of steps served as the dependent variable, this educational protocol was independently associated with a higher number of steps [coefficient 988 steps, 95% CI 137–1838, *p* = 0.02], as was smoking, after adjustment for surgical length, emergent surgery, maternal age and body mass index.

**Conclusion:**

An educational protocol which included earlier ambulation and regular interval pain control was associated with improved ambulation following CD.

## Background

Thromboembolic events are among the leading causes of maternal mortality in developed countries [1]. The physiological changes of pregnancy are associated with an increased risk of venous thromboembolism (VTE), which peaks in the immediate postpartum period [2], but may persist for up to 12 weeks following delivery [3]. Past studies have identified risk factors for postpartum VTE, such as advanced maternal age, Afro-American ethnicity, obesity, infection and thrombophilia [4, 5]. In addition, in a large scale meta-analysis, VTE was found fourfold more common following caesarean delivery (CD) as compared to vaginal delivery, more so following emergent surgery [6].

Immobility, which characterizes the immediate post-partum period, has been previously demonstrated to contribute to the pathogenesis of VTE [7]. Previous studies have addressed post-partum immobilization, and linked it to an increased risk for VTE [8], yet only a limited number have objectively quantified mobility with digital step counters [9–11]. Moreover, none of the previous studies have assessed the efficacy of an intervention for reducing post-partum immobilization.

Pregnancies are now postponed and achieved at a more advanced age, obesity is more common than ever in developed countries, and multiple gestation rates rising due to assisted reproduction use. In light of these changes in patient demographics over the years and the increase in CD rates [12], the anticipated rate of at-risk patients for thromboembolic events following delivery is anticipated to rise further. There is a lack of uniformity between institutions regarding different protocols for VTE risk reduction, and current protocols rely on non-obstetric literature regarding post-surgical care and empiric practice. Thus, we focused on areas of care with a potential for further improvement – staff and patient education, ambulation timing ang analgesic control, and investigated the efficacy of a new protocol for the enhancement of postpartum mobility following CD. Mobility was assessed with digital step counters, an objective and reliable means of measurement, lacking in previous studies.

## Methods

This was a prospective interventional study conducted at the maternity unit of the Edith Wolfson Medical Center between June 2017 and July 2018.

### Patients

Patients who underwent a CD during that period were included. Excluded were patients restricted to bed rest following CD, such as those evaluated to be at high risk of falling, hemodynamically unstable or those who necessitated continuous intravenous treatment for the first 24 h following delivery (such as magnesium-sulphate).

### Data collection

Patients who underwent CD were offered participation in the study by an obstetrician from our research team, while in the recovery room after surgery. As the study objective was to assess the effect of a new protocol on patient mobility, pedometers were determined the most objective and reliable means of measurement. Those who consented were asked to wear a pedometer (*HI-TEC*, China) around their wrist for the immediate 48 h following delivery. This time frame was chosen as it is associated with a higher tendency for immobility and higher thromboembolic risk. After 48 h, our research team removed the pedometers, and asked the patients if the pedometer had been worn the entire 48 h, and not removed. If it has been removed, patient data was excluded. Patients were not required to operate their pedometers as they were preset to record the number steps taken. Pedometers were examined periodically to detect any malfunctions by our research team, and measurements were compared and validated with a smart phone application.

### The previous departmental protocol

From June 2017 to February 2018, we assessed the effect of our previous departmental protocol on patient mobility. As part of the previous departmental protocol, upon admission to the maternity unit, all patients were evaluated by the attending nurse, and ambulation initiated 6 h following delivery, initially to an assistant seating position in a couch. Urinary catheter was also removed 6 h following surgery. At this point, patients resumed gradual ambulation and intake. Intravenous (IV) acetaminophen (one gram) was administered in a scheduled treatment protocol, every 6 h for the first 24 h following delivery, during which the patient could also request additional medication for pain (breakthrough medication). Following 24 h, pain medications were given only upon patients’ request, according to the Visual Analogue Scale (VAS) assessment for pain, performed routinely by the department nurses. For a VAS score of 4–7, or persistent pain which did not resolve 1 h after administration of acetaminophen – dipyrone one gram was administered (maximum four grams daily), while for a VAS of 8–10, or persistent pain which did not resolve 1 h after administration of dipyrone - ibuprofen 400 mg was administered (maximum 1200 mg daily).

### Investigational protocol period

**Fig. 1:**
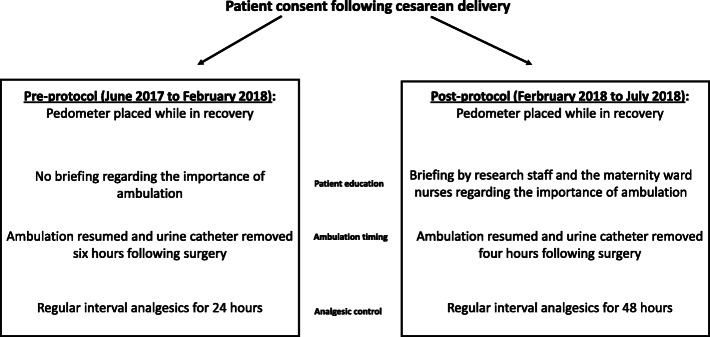
Main characteristics of the pre-protocol and post-protocol periods of the study

From February 2018 to July 2018, we assessed the effect of newly designed educational protocol, which consisted of patients’ education and nurses’ tutoring, regarding the importance of early ambulation. Nurses were briefed by the head researcher (author H.G.H.) and by the department head (author M.K.), regarding the anticipated changes in protocol. They were presented with current statistics regarding thromboembolic complications and their peak incidence in the first week postpartum and following CD. The nurses were also instructed regarding the importance of patient ambulation during hospitalization. They were asked to incorporate this information while briefing patients at admission to the department. All participants were also briefed by one of our research staff regarding the study objective and the importance of ambulation. They were also given a written page with this information. Upon admission to the maternity unit, all patients were examined by the attending nurse and ambulation was performed 4 h after surgery. Patients were treated with one-gram IV acetaminophen, at 6-h intervals for 48 h (compared to 24 h prior). Patients could also request additional medication for pain as needed (breakthrough medication), according to VAS assessment, as specified. (Fig. 1).

Thrombophylaxis treatment (Enoxaparin) was given based on patient’s risk factors, such as maternal age and obesity. Patients in the maternity ward may choose for their infant to be with them (“homing”), or in the nursery. All patient rooms are located approximately 50 ft from the nursery.

### Comparison of two groups of patients

We compared two groups of patients, prior to the implementation of the investigational protocol (pre-protocol group) and following implementation of the protocol (post-protocol group).

We collected data regarding maternal characteristics, surgical and postpartum course, with intent to compare it between the groups. Demographic characteristics were collected from patient files, and included maternal age, body mass index (BMI kg/m^2^) – both pre-gravid and at delivery, gravidity, parity, prior CDs, comorbidities and active smoking during pregnancy. Labor course was reviewed and complications noted, including fever and antibiotic treatment. Maternity ward hospitalization charts were reviewed for complications, including fever and or blood transfusions, pain assessments and analgesic consumption, breastfeeding and length of hospitalization. To account for a potential effect of enhanced mobility immediately following CD on surgical wound complications and thromboembolic events - a telephone survey was performed two to three months following delivery and patients were questioned regarding any late complication (which may have been treated at a different institution)..

### Statistical analysis

Data were analyzed with Epi Info, version 7.0 (Centers for Disease Control and Prevention, Atlanta, GA). Continuous variables were calculated as mean ± standard deviation or median and range as appropriate and compared with the use of the Student t-test or Mann-Whitney test. Categorical variables were calculated as number (%) and compared using the Chi-square test or Fisher’s exact test as appropriate. All tests were two sided, and a *p* value of less than 0.05 was considered statistically significant.

Patient enrollment was continued until at least 100 patients in each group completed their participation, and wore the pedometer as required. Sample size calculation targeted the lower mobility patients for two considerations: first, the authors’ hypothesis was that the lowest mobility patients would be at the highest risk for thromboembolism. We assumed these patients would benefit most from intervention, and that an intervention aimed at the highest mobility patients may not be as beneficial, in terms of thromboembolism risk reduction (disregarding additional benefits following surgery, such as bowel movement resumption). Second, no data was available to us apriori to study initiation regarding the average number and standard deviation of steps 48 h following cesarean delivery. We therefore aimed for the lower quartile of steps in the pre-protocol group to represent the lower 10th percentile of steps of the post-protocol, so that the rate of lowest mobility patients pre-protocol would be diminished. This required a sample size of 100 patients in each group. Thus, 100 patients were recruited prior to the new protocol implementation, and from February 2018 at which time the new protocol was introduced, we recruited an additional 100 patients. Analysis was performed by the intention to treat principle.

### Ethical considerations

The study was approved by the Edith Wolfson Medical Center institutional review board, approval number #0230–16-WOMC, and informed consent was obtained from all patients who participated, by signing an Informed Consent Form.

## Results

### Patient enrollment

**Fig. 2:**
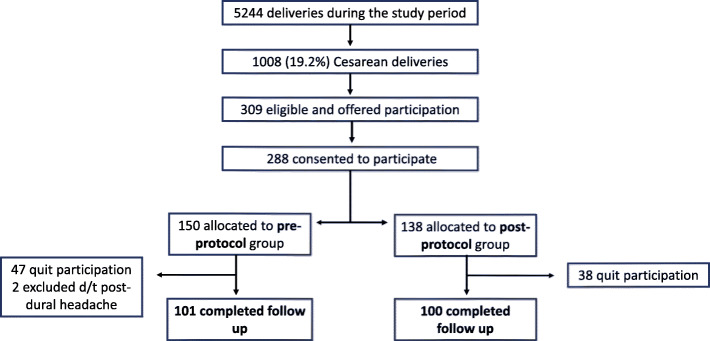
Flow chart of patient recruitment and participation

During the study period between June 2017 and July 2018, 309 patients were offered participation in the study. Twenty-one patients (6.7%) declined, and 288 patients (93.2%) consented to participation. Among patients who consented, two were excluded due to post-dural headaches following epidural anesthesia necessitating bed rest, and 85 patients removed their pedometer during follow up. A total of 201 patients (69.7%) completed their follow and were included in the analysis, 101 in the pre-protocol group and 100 in the post-protocol group. (Fig. 2)

### Patient demographics

Patient demographics were not significantly different between the study groups, including patient age, BMI, gravidity, parity, number of prior CDs and smoking (Table 1). The rate of multiple gestation, preeclampsia and diabetes mellitus were also non-significant between the groups.

**Table 1:** Demographic characteristics of the pre-protocol and post-protocol groups

	Pre-protocol group *n* = 101	Post-protocol group *n* = 100	P
Age, (years), mean ± SD	33.0 ± 5.3	32.2 ± 5.5	0.26
BMI^a^ (kg/m^2^) – pre-gestational, mean ± SD	24.5 ± 5.0	25.5 ± 5.7	0.21
BMI^a^ (kg/m^2^) – at delivery, mean ± SD	29.6 ± 4.8	30.6 ± 5.3	0.17
Gestational weight gain, (kg), mean ± SD	13.3 ± 6.1	13.1 ± 5.4	0.81
Assisted reproduction, n (%)	22 (21.7%)	14 (14.0%)	0.15
Gravidity, median (range)	3 (1–8)	3 (1–8)	0.82
Parity, median (range)	1 (0–5)	1 (1–7)	0.66
Prior CDs^b^, median (range)	1 (0–4)	1 (0–5)	0.43
Multiple gestation, n (%)	9 (8.9%)	11 (11.0%)	0.62
Hypertension^c^ / Preeclampsia, n (%)	2 (1.9%)	3 (3.0%)	0.68
Diabetes mellitus^d^, n (%)	11 (10.8%)	10 (10.0%)	0.83
Smoking, n (%)	28 (27.7%)	17 (17.0%)	0.06

^a^*BMI* body mass index; ^b^*CD* cesarean delivery; ^c^Hypertension – chronic or gestational; ^d^Diabetes mellitus – pre-gestational or gestational

### Intrapartum characteristics

Gestational age was non-significantly different between the pre and post protocol groups (Table 2). The rate of primary CDs was 43.5 and 49.0% in the pre and post-protocol groups, respectively, *p* = 0.43. Surgical characteristics also did not differ between the groups, including: intraoperative adhesions (of any degree), estimated blood loss and additional surgical procedure (tubal ligation or cystectomy). Surgeries in the pre-protocol group were slightly shorter as compared to the post-protocol group - 48.5 ± 14.6 vs. 53.5 ± 15.3 min, *p* = 0.02. The rate of intrapartum complications (chorioamnionitis, blood transfusion) was non-significant between the groups.

**Table 2:** Labor course of the pre-protocol and post-protocol groups

	Pre-protocol group *n* = 101	Post-protocol group *n* = 100	P
Gestational age, (weeks), mean ± SD	38.1 ± 2.1	38.2 ± 1.7	0.66
Emergent CD^a^, n (%)	50 (49.5%)	50 (50.0%)	0.94
Primary CD, n (%)	44 (43.5%)	49 (49.0%)	0.43
Adhesions^b^, n (%)	29 (28.7%)	35 (35.0%)	0.33
Operative length, (minutes), mean ± SD	48.5 ± 14.6	53.5 ± 15.3	0.02
Estimated blood loss, (ml), mean ± SD	674 ± 175	703 ± 281	0.39
Additional surgical procedure, n (%)	9 (8.9%)	8 (8.0%)	0.81
Intra-partum complications, n (%)	5 (4.9%)	3 (3.0%)	0.72

^a^*CD* cesarean delivery

^b^Adhesions – any adhesions, as reported by surgeon in surgical report; Additional surgical procedure – tubal ligation or cystectomy

### Post-partum course and study outcomes

A non-significant rate of postpartum complications occurred in both study groups (Table 3). Notably, patients in the post-protocol group consumed less additional analgesics - median 4 doses, range 1–10 vs. median 6 doses, range 0–11, *p* < 0.001. All patients were discharged an average 4 days following surgery. The average number of steps taken following delivery was significantly higher in the post-protocol group as compared to the pre-protocol one, 4394 ± 2985 vs. 3551 ± 2931 steps, respectively *p* = 0.04.

**Table 3:** Post-labor course of the pre-protocol and post-protocol groups

	Pre-protocol group *n* = 101	Post-protocol group *n* = 100	P
Post-partum complication^a^, n (%)	5 (4.9%)	5 (5.0%)	0.98
Hemoglobin decrease, (dL/g), mean ± SD	0.9 ± 1.7	1.1 ± 1.0	> 0.99
Postpartum hemoglobin < 10 dL/g, n (%)	23 (22.7%)	27 (27.0%)	0.48
S.O.S^b^ medication consumption, median (range)	6 (0–11)	4 (1–10)	< 0.001
Breastfeeding^c^, n (%)	67 (66.3%)	63 (63.0%)	0.62
Homing^d^, n (%)	1 (0.9%)	3 (3.0%)	0.36
Hospitalization, (days), mean ± SD	4.0 ± 0.5	4.1 ± 0.8	0.74
Clexane treatment, n (%)	1 (0.9%)	5 (5.0%)	0.11
Steps^e^ (total), mean ± SD	3551 ± 2931	4394 ± 2985	0.04

^a^Postpartum complication – endometritis/ wound infection/ blood transfusion; ^b^S,O.S medication – analgesics given on-demand (in addition to regular interval analgesics); ^c^Breastfeeding – exclusively, no formula supplementation; ^d^Homing – neonate at mother’s care in her room (as opposed to nursery care); ^e^Steps – number of steps taken by patients, as recorded by pedometer

### Regression analysis

A linear regression analysis was performed, in which the total number of steps taken served as the dependent variable, and implementation of the protocol, patient age, BMI, smoking, emergent surgery and surgical length served as independent variables (Table 4). Protocol implementation - CE 988.2, 95%CI 137.5–1838.8, *p* = 0.02, and smoking - CE 1640.4, 95%CI 621.3–2659.4, *p* = 0.001, were found independently associated with steps taken, after adjustment for confounders.

**Table 4:** Linear regression model for number of steps following delivery

	Coefficient	95% C.I^a^		P
		Lower	Upper	
Protocol implementation	988.2	137.5	1838.8	0.02
Age, years	−65.9	− 146.7	14.9	0.10
Body mass index, kg/m^2^	76.8	−4.3	158.1	0.06
Smoking	1640.4	621.3	2659.4	0.001
Emergent surgery	482.9	− 374.0	1339.8	0.26
Operative length	4.9	−24.7	34.6	0.74

^a^*CI* confidence interval

We performed a telephone survey, and none of the participants reported thromboembolic complications. One was admitted and treated with parenteral antibiotics for suspected endometritis 1 week following delivery, one reported oral antibiotic treatment subscribed for suspected wound infection and one underwent hysteroscopic removal of retained products of conception.

## Discussion

We conducted this study to investigate the efficacy of an improved post-partum protocol for the enhancement of patient mobility following CD. We implemented a new protocol, which included patient education and nurses tutoring, earlier ambulation of patients following surgery, and extended the use of regular interval IV analgesics (48 instead of 24 h). This resulted in an increased number of steps taken by patients and a reduction of breakthrough medication consumption, with no additional immediate or delayed post-partum complications.

In an effort to halt maternal mortality in the developed world, thromboembolic events are an area of focus, including timely identification of risk factors, and potential interventions to reduce this risk. Maternal age, BMI, parity, multiple gestation, diabetes, preeclampsia and emergent CD have all been described as risk factors for VTE following delivery, and these serve as indications for medical thrombophylaxis following delivery, according to the American College of Obstetricians and Gynecologist [13]. Similarly, the Royal College of Obstetricians and Gynecologists recommends that all patients be treated with low molecular weight heparin for 10 days following CD, with the exception of elective surgeries, for which treatment is required in the presence of an additional risk factor [14]. Finally, according to European guidelines [15] and the American College of Chest physicians [16], the decision to initiate treatment relies on an initial assessment of patient mobility, and can be avoided in low risk patients with early rehabilitation.

All above recommendations stress the importance of proper postpartum mobilization. The accuracy and reliability of activity trackers in the assessment of postpartum mobility has been recently verified, and correlated to quality of recovery [17]. Yet, a limited number of studies have assessed postpartum mobility with their use. In a study by Ma and colleagues, patient mobility during the first 24 h following delivery was assessed with activity trackers [10]. They demonstrated an increased mobility following vaginal delivery as compared to CD, despite similar analgesic consumption. In an additional study by Sharma et al., patients were followed for a week after delivery [11]. Similarly to the study by Ma et al., patients who delivered vaginally were mobile sooner and were more active during their follow up. Interestingly, patients following an emergent CD were more mobile than those following an elective one. Our group has also recently described our findings of an observational study which assessed the number of steps taken during the first 48 h following delivery [9]. We failed to demonstrate any patient or surgical variables associated with reduced ambulation, although smoking was found independently associated with enhanced ambulation. This finding was reinforced in the current study, and probably relates to the need to ambulate outside the ward to smoke. Despite all previous mentioned studies, none were interventional, and thus the need for the current study arose.

Despite different recommendations for the prevention of VTE following CD, the study was undertaken due to lack of uniformity of institutional practices and lack of obstetric literature regarding post CD care. While improving the protocol for postpartum care of patients following CD, we considered three points which could potentially hamper patients’ mobility: first, lack of awareness to the importance of mobility by patients and caregivers. This point was approached in an acceptable manner to both staff and patients, and required focused nurse training, and a patient education upon admission to the department. Second, lack of ability to ambulate earlier - in the pre-protocol period, the departmental policy for mobilization of patients following CD relied mostly on recommendations from other surgeries, as no trial had assessed the effect of different time frames for mobilization following CD. The nurses in the pre-protocol period initiated such ambulation 6 h following delivery, in accordance with our department protocol. However, after careful literature search while planning the study, we found no reason to wait this time, as many patients will inquire if they can ambulate earlier, and ambulating earlier offers the opportunity for overall enhanced mobility. Eventually, during our study four patients did not wish to ambulate earlier following delivery, but earlier ambulation was acceptable and even welcome by all others. In other words, it is safe to assume that when offered, the majority of patients would prefer earlier ambulation. Finally, we considered the effect of improper pain control on patient immobilization. Previous trials have assessed the optimal medical combination for proper pain control, with patient pain scores and breakthrough medications as the main outcome employed [18, 19]. This did not allow for an extrapolation of previous data for our study objective, but we assumed fixed interval doses for an extended time would yield improved pain control and enhanced mobility [20]. This point was validated by the decreased consumption of additional analgesics in the post-protocol group, and points to patient mobility as an additional study objective in future trials assessing different postpartum analgesic protocols. Overall, while the current study design does not allow for an individual assessment of the contribution of each component, the authors considered all three necessary for enhanced ambulation.

Our study is not without limitations. First, despite its prospective nature, the study was not randomized. Due to the overall limited study period, patient characteristics were similar between the pre and post-protocol groups. The exception was surgical length which was slightly higher in the post protocol group, despite similar surgical practices throughout the study. We did account for surgical length in the logistic regression analysis performed, to minimize the effect of this difference. Nevertheless, a future study design would optimally be randomized, and may also be separated to evaluate each of the individual changes to protocol independently. Second, 85 of 286 patients (29.7%) were excluded from analysis because they removed their pedometer. We attempted to minimize the potential selection bias by comparing patient demographics and delivery course between patients lost to follow up and those included, and found no difference. We are aware this could imply to the generality of our results. Third, while planning the study, we considered the possibility that enhancing mobility among patients who properly ambulate following delivery is not necessary, and perhaps harmful. However, in the current study, no excess risk of immediate or delayed postpartum complications was noted in the postpartum group, who also consumed less analgesics. Moreover, the study sample was calculated with the objective of improving mobility among the lowest mobility patients, and indeed - the lower quartile of steps in the pre-protocol group (1417 steps) represented the lower 10th percentile of steps post-protocol (1478 steps), so that the rate of lowest mobility patients pre-protocol was diminished by more than half. Finally, patient satisfaction was not evaluated, and should be assessed in future studies.

Despite the limitations noted, this is the first study to the author’s knowledge to investigate the effect of an intervention on the postpartum mobility of patients. We also used an objective means of measurement – digital step counters, and focused on an at-risk population of patients following CD.

## Conclusions

This study has demonstrated that earlier ambulation and proper pain control can improve patients’ mobility following CD, by proper staff and patient education. As VTE is among the leading causes of maternal death in the developed world, it is highly important to focus on high risk patients following CD. Future large-scale studies will be necessary to determine the effect of different interventions on the occurrence of VTE.

## Data Availability

The data used and/or analyzed during the current study are available from the corresponding author on reasonable request.

## Abbreviations

CD: Cesarean delivery
IV: Intravenous
VTE: Venous thrombo-embolism
VAS: Visual analogue score
BMI: Body mass index
CE: Coefficient
CI: Confidence interval
